# Body composition patterns among type 2 diabetes mellitus patients versus nondiabetic adults in Saudi Arabia

**DOI:** 10.3389/fendo.2025.1494452

**Published:** 2025-06-18

**Authors:** Eman Alfadhli, Ishraq Darandari, Maha Altaweel, Sara Alharbi, Asma Jadw, Ghadi Aljohani, Sarah Mohammad

**Affiliations:** ^1^ Medicine Department, College of Medicine, Taibah University, Medina, Saudi Arabia; ^2^ Medicine Department, King Faisal Specialist Hospital & Research center, Medina, Saudi Arabia

**Keywords:** type 2 diabetes mellitus, body composition, bioelectrical impedance analysis, obesity, body fat, visceral fat

## Abstract

**Objectives:**

To explore differences in body composition between individuals with type 2 diabetes mellitus (T2DM) and those without diabetes in Medina, Saudi Arabia, stratified by sex and age.

**Methods:**

A cross-sectional study was conducted at Taibah University, four primary care centers, and diabetes center in Medina, Saudi Arabia, from July to September 2023, involving 630 adults with and without T2DM. Body composition was assessed using a bioelectrical impedance analysis (BIA), measuring weight, body mass index (BMI), total body fat, visceral fat (VF), muscle mass, and bone mass. Participants were grouped into three categories: young age (18–40 years), middle age (41–60 years), and older age (>60 years). Body composition differences between groups were analyzed using independent t-tests.

**Results:**

Of the 630 participants, 42.4% had T2DM. Among young women with T2DM, BMI, total body fat, VF, muscle mass, and bone mass were significantly higher (p < 0.001) compared to women without diabetes. However, their muscle and bone mass percentages were lower. In contrast, no significant differences were found between middle-aged women with and without T2DM. Among older women, those with T2DM had significantly higher BMI (p = 0.030) and VF (p = 0.007). For men, body composition differences were mostly non-significant across age groups, except for lower muscle mass percentage in young men with T2DM (p = 0.013).

**Conclusion:**

Sex- and age-specific differences in body composition exist between adults with and without T2DM. These findings highlight the importance of tailored strategies in T2DM prevention and management. Future research should examine underlying mechanisms and evaluate the impact of targeted interventions.

## Introduction

Type 2 diabetes mellitus (T2DM) is a chronic metabolic disorder closely linked to obesity, a major public health concern and a primary contributor to the disease (1, 2). The global prevalence of both obesity and T2DM has been rising at an alarming rate (3). This interrelationship is often referred to as “diabesity,” highlighting the complex and bidirectional nature of the two conditions (4–6). A key factor driving this association is body composition, particularly the accumulation of visceral fat (VF), which is strongly associated with insulin resistance and the pathogenesis of T2DM (7, 8).

Obesity is often assessed using metrics like body mass index (BMI) and waist circumference (WC). While useful, these metrics are limited in their ability to differentiate between fat mass and lean mass, which may obscure important metabolic differences in individuals with T2DM (9). As a result, more advanced methods such as body composition analysis are used to provide a clearer distinction between fat and lean tissue distribution (10).

Among body composition assessment tools, dual-energy X-ray absorptiometry (DXA) is often considered one of the most comprehensive imaging techniques among non-invasive methods (9). However, its high cost and limited accessibility make it less feasible for routine use in large-scale studies or clinical practice. Bioelectrical impedance analysis (BIA), by contrast, offers a cost-effective, non-invasive, and widely available alternative for assessing key body composition indicators such as total body fat, VF, muscle mass, and bone mass (10, 11). BIA has proven especially valuable in population screening and large-scale epidemiological studies due to its simplicity and speed (12).

Despite the close association between body composition and T2DM, few studies have investigated body composition patterns among individuals with T2DM, particularly in in Middle Eastern populations (13–15). Moreover, the influence of sex and age on these patterns remains underexplored, leaving a gap in understanding body composition differences in T2DM (16–18). Therefore, this study aims to investigate body composition differences among adults with and without T2DM in Medina, Saudi Arabia, using BIA, while stratifying by sex and age group. Understanding variations in body fat and lean mass may help tailor lifestyle or pharmacologic interventions according to patient demographics, ultimately improving diabetes prevention and management strategies.

## Methods

This cross-sectional study included 630 adults recruited from Taibah University, four primary care centers, and the Diabetes Center in Medina, Saudi Arabia. Participants were recruited through announcements and direct invitations at the participating centers. Non-T2DM individuals were recruited from the same centers, including staff members, patient companions, and those attending for routine health check-ups. While this method may introduce selection bias, particularly among non-T2DM individuals, efforts were made to include participants of diverse ages and both genders to enhance sample variety. The study was conducted between July and September 2023. Ethical approval was granted by the Research Ethics Committee of Taibah University, College of Medicine, Medina, Saudi Arabia (Approval Code: STU-22-002), and informed consent was obtained from all participants.

Eligible candidates were Saudi individuals, aged 18 and older, with or without T2DM and willingness to participate. Exclusion criteria included individuals with type 1 diabetes, pregnancy, severe comorbidities, including cancer, acute illness, and chronic liver, kidney, or heart disease. Individuals on steroid medications, dietary supplements, or those engaged in athletic or physically demanding occupations were excluded from the study.

To screen for undiagnosed T2DM among non-T2DM participants, capillary blood glucose measurements were obtained from fasting or random finger-prick samples. While laboratory-based venous plasma glucose testing is the gold standard for definitive diagnosis, capillary blood glucose testing offers a rapid and convenient method for initial screening, albeit with some limitations in accuracy. Participants meeting the American Diabetes Association’s (ADA) diagnostic criteria for diabetes (19), including fasting glucose levels ≥126 mg/dL or random plasma glucose levels ≥200 mg/dL, underwent confirmatory Hemoglobin A1c (HbA1c) testing. The HbA1c testing was performed using standard laboratory procedures, with a cut-off value of 6.5% to diagnose T2DM, in line with the ADA guidelines (19). Individuals with confirmed T2DM were excluded from the study.

Data collection involved recording demographic information (age, sex, and exercise frequency). For participants with T2DM, additional information was gathered on diabetes duration, hypoglycemic medication usage, and the most recent HbA1c levels, sourced from self-reports and medical records.

Body composition was assessed using the Eufy body composition analyzer (Model T9147, P1, the full-body Smart Scale, Anker Technology Ltd, Birmingham, United Kingdom) which estimates weight, BMI, fat mass, and muscle mass via BIA. Although validation studies for this specific model are limited, similar consumer-grade BIA devices have shown acceptable correlation with DXA in previous research (10). Body composition measurements were taken from participants at least two hours after the last food intake and exercise to minimize the effects on measurement accuracy. Participants wore light clothing and removed any metallic items to avoid interference with the measurements.

Participants were categorized into three age groups: young adults (18–40 years), middle-aged adults (41–60 years), and older adults (>60 years) These age groups were chosen to align with common demographic classifications and to capture potential age-related differences in body composition. Body composition data were analyzed to compare differences between individuals with and without T2DM, considering sex and age group.

### Statistical analysis

The Statistical Package for the Social Sciences, version 26.0 (SPSS Inc., Chicago, IL) was used to analyze the data. Normality of continuous variables was assessed using the Kolmogorov–Smirnov test and visual inspection via histograms and Q-Q plots. Continuous data were presented as mean and standard deviation (SD), whereas categorical variables were represented as frequencies and percentages. Categorical variables were analyzed using the Chi-square test, and Fisher’s exact test was used to determine whether there was a significant association between body compositions and T2DM across age groups and sex. Independent t-tests were used to calculate differences in body composition between T2DM and non-T2DM by age group and sex. A P-value of less than 0.05 was considered statistically significant. No adjustments were made for multiple comparisons; therefore, results should be interpreted with caution due to the increased risk of Type I error.

## Results

A total of 633 individuals were initially enrolled in the study. After screening, three individuals from the non-T2DM group were newly diagnosed with T2DM and excluded. The final sample comprised 630 participants: 42.4% with T2DM and 57.6% without T2DM. Of these, 257 (40.8%) were male and 373 (59.2%) were female. **Table 1** presents the baseline demographic and clinical characteristics by T2DM status. The participants had a mean age of 48.5 ± 12.7 years, and 58.3% of participants fell into the middle-age group. Statistical analysis revealed no significant differences in mean age between individuals with and without T2DM within each age group (p values > 0.05). Also, there were no significant differences in exercise frequency between T2DM and non-T2DM groups (p = 0.19), as shown in **Table 1**.

**Table 1:** Baseline demographic and clinical characteristics of participants by T2DM status.

	Characteristics	All participants 630	Diabetes status		P-value
			Non-T2DM 363 (57.6%)	T2DM 267 (42.4%)	
Sex	Male/Female	257 (40.8%) /373 (59.2%)	150 (58.4%) /213 (57.1%)	107 (40.6% /160 (42.9%)	0.753
Participants in each age group: N (%)					
Young-Age Group (18–40 years)		164 (26.0%)	135 (82.3%)	29 (17.7%)	
Middle-Age Group (41–60 years)		367 (58.3%)	182 (49.6%)	185 (50.4%)	
Older-Age Group (>60 years)		99 (15.7%)	46 (46.5%)	53 (53.5%)	
Mean age of each age group (years)					
Young-Age Group (18–40 years)		31.8 (6.0)	31.4 (5.7)	33.7 (7.0)	0.057
Middle-Age Group (41–60 years)		50.9 (5.5)	50.1 (5.4)	51.7 (5.4)	0.060
Older-Age Group (>60 years):		67.2 (5.3)	67.5 (6.3)	66.9 (4.3)	0.632
Exercise frequency (%)					
none		246 (39.0%)	151 (41.6%)	95 (35.6%)	0.190^^^
On rare occasion		113 (17.9%)	63 (17.4%)	50 (18.7%)	
Few times/month		65 (10.3%)	32 (8.8%)	33 (12.4%)	
1–4 times/week		115 (18.3%)	71 (19.6%)	44 (16.5%)	
5–7 times/week		91 (14.4%)	46 (12.7%)	45 (16.9%)	
High body mass index categories: N (%)					
Overweight (BMI 25-29.9%)		206 (32.7%)	125 (34.4%)	81 (30.3%)	0.002*#
Obese (BMI ≥ 30%)		276 (43.8%)	137 (37.7%)	139 (52.1%)	
Overweight & Obese (BMI ≥ 25%)		482 (76.5%)	262 (72.1%)	220 (82.4%)	

Data are presented as means ± SD, or as frequencies (%). T2DM—type 2 diabetes mellitus, BMI—body mass index. *P-value is statistically significant. ^This p-value corresponds to the overall chi-square test for all Exercise frequency categories. ^#^This p-value corresponds to the overall chi-square test for all BMI categories.

**Figure 1:**
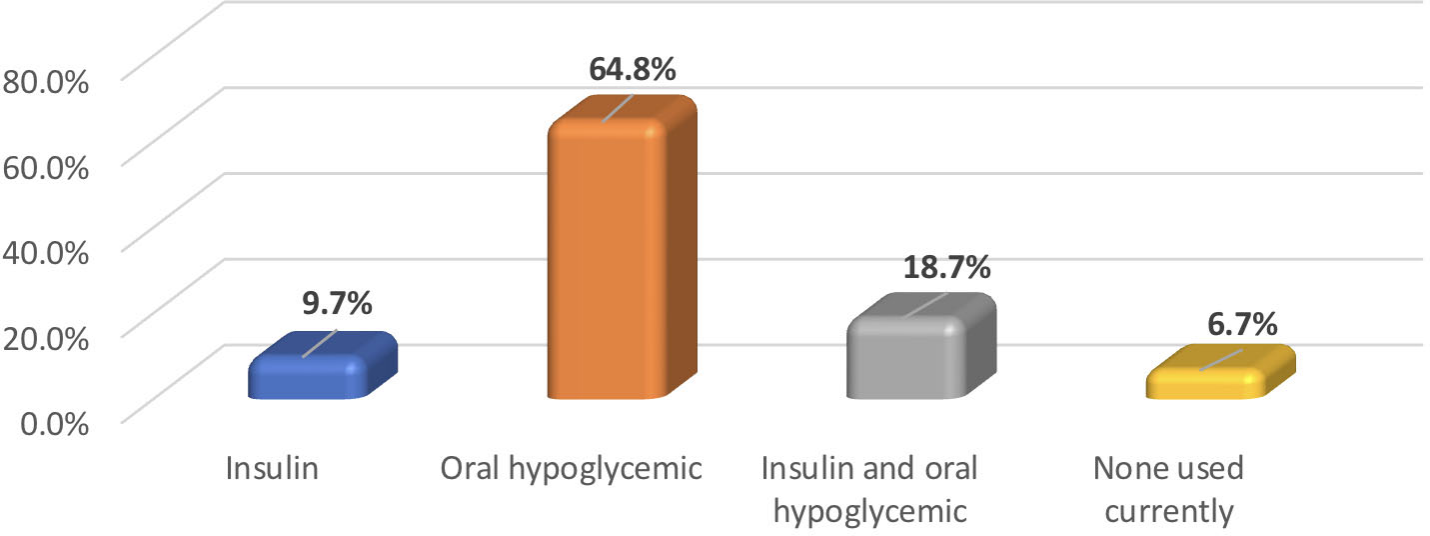
Frequency of hypoglycemia treatment modalities utilized by participants with T2DM (N = 267).

Among T2DM group, the mean diabetes duration was 9.6 ± 7.8 years, and the mean HbA1c level was 8.1% ± 1.8. The frequency of treatment modalities among individuals with T2DM are provided in **Figure 1**.

Overall, individuals with T2DM exhibited higher rates of obesity; mean BMI (29.6 ± 4.3 kg/m²) compared to non-T2DM individuals (27.8 ± 3.9 kg/m²); p < 0.001). Sex and age were pivotal factors associated with differences in body composition, as visually summarized in **Table 2** which presents body composition parameters stratified by age group and sex.

**Table 2:** Comparison of body composition between participants with or without T2DM stratified by age group and sex.

Age group (years)		18-40 (*n*= 178)			41-60 (*n*=367)			> 60 (*n*=99)		
Body composition	Sample	Non-T2DM (N=135)	T2DM (N=29)	P-value	Non-T2DM (N=182)	T2DM (N=185)	P-value	Non-T2DM (N= 46)	T2DM (N=53)	P-value
Body mass index	All	26.6 (± 5.7)	32.94 (± 6.4)	<.001*	29.87 (± 5.9)	30.5 (± 6.4)	0.334	27.93 (± 4.9)	29.81 (± 4.9)	0.062
	Female	26.73 (± 6.3)	36.65 (± 5.1)	<.001*	30.17 (± 6)	31.56 (± 6.8)	0.123	28.02 (± 4.8)	31.13 (± 5.3)	0.030*
	Male	32.10 (± 5.1)	29.48 (± 5.5)	0.05	29.55 (± 5.8)	28.63 (± 5.3)	0.308	27.82 (± 5.2)	28.32 (± 4.2)	0.716
Body fat (%)	All	31.53 (± 10.7)	35.62 (± 11.5)	0.067	33.94 (± 9)	35.5 (± 9.6)	0.108	32.69 (± 8.7)	34.34 (± 8.9)	0.355
	Female	35.38 (± 9)	45.83 (± 3.1)	<.001*	39.77 (± 6.6)	40.41 (± 7.4)	0.517	38.01 (± 6.4)	40.76 (± 6.3)	0.122
	Male	21.99 (± 7.9)	26.09 (± 7.4)	0.078	27.71 (± 6.7)	26.86 (± 6.3)	0.423	26.35 (± 6.7)	27.15 (± 4.9)	0.643
Body fat (kg)	All	22.52 (± 10.5)	32 (± 13.3)	<.001*	28.13 (± 11.3)	29.36 (± 12)	0.312	24.25 (± 9.1)	27.68 (± 9.9)	0.085
	Female	24.46 (± 11.2)	42.38 (± 9.3)	<.001*	31.13 (± 10.8)	32.73 (± 11.9)	0.311	26.08 (± 9.3)	31.12 (± 9.3)	0.055
	Male	17.94 (± 9.3)	22.31 (± 8.2)	0.111	24.93 (± 11.1)	23.43 (± 9.6)	0.379	22.08 (± 8.6)	23.64 (± 9.1)	0.554
Visceral fat (rating)	All	7.68 (± 3.7)	11.66 (± 2.8)	<.001*	11.81 (± 3.9)	11.81 (± 3.8)	0.998	11.95 (± 4.3)	13.77 (± 3.5)	0.022*
	Female	6.47 (± 3.2)	11.07 (± 2.2)	<.001*	9.3 (± 2.9)	10.15 (± 3.5)	0.057	9.32 (± 2.6)	11.29 (± 2.5)	0.007*
	Male	10.02 (± 3.9)	12.2 (± 3.3)	0.058	14.49 (± 3)	14.73 (± 2.4)	0.599	15.09 (± 3.7)	16.56 (± 2)	0.115
Muscle mass (%)	All	62.55 (± 9.8)	57.97 (± 10.6)	.026*	60.42 (± 8.3)	59.12 (± 8.8)	0.146	60.93 (± 7.9)	60.14 (± 8.0)	0.623
	Female	58.86 (± 7.9)	49.8 (± 2.7)	<.001*	54.97 (± 5.8)	54.6 (± 6.6)	0.672	56.62 (± 5.6)	54.33 (± 5.6)	0.143
	Male	71.70 (± 7.7)	65.59 (± 9.5)	0.013*	66.24 (± 6.3)	67.08 (± 6)	0.402	66.07 (± 7.2)	66.65 (± 4.5)	0.738
Muscle mass (kg)	All	42.36 (± 9.2)	50.68 (± 6.9)	<.001*	48.08 (± 10)	46.83 (± 9)	0.209	44.41 (± 9)	46.04 (± 8.7)	0.361
	Female	37.57 (± 5.3)	45.46 (± 5.7)	<.001*	40.79 (± 5.9)	41.89 (± 6.3)	0.196	37.84 (± 4.9)	39.74 (± 5)	0.171
	Male	53.92 (± 6.1)	55.55 (± 3.6)	0.281	55.86 (± 7.1)	55.53 (± 6)	0.757	52.22 (± 5.8)	53.09 (± 6.1)	0.627
Bone mass (%)	All	3.71 (± 0.4)	3.41 (± 0.4)	<.001*	3.49 (± 0.3)	3.45 (± 0.3)	0.245	3.49 (± 0.3)	3.43 (± 0.3)	0.301
	Female	3.66 (± 0.4)	3.2 (± 0.2)	<.001*	3.41 (± 0.3)	3.36 (± 0.3)	0.250	3.36 (± 0.3)	3.3 (± 0.2)	0.359
	Male	3.85 (± 0.4)	3.61 (± 0.4)	0.056	3.58 (± 0.3)	3.61 (± 0.3)	0.525	3.65 (± 0.3)	3.58 (± 0.2)	0.407
Bone mass (kg)	All	2.52 (± 0.4)	2.94 (± 0.2)	<.001*	2.77 (± 0.4)	2.73 (± 0.4)	0.370	2.52 (± 0.4)	2.63 (± 0.4)	0.196
	Female	2.36 (± 0.4)	2.91 (± 0.3)	<.001*	2.54 (± 0.4)	2.59 (± 0.4)	0.420	2.26 (± 0.4)	2.45 (± 0.4)	0.064
	Male	2.90 (± 0.3)	2.97 (± 0.2)	0.330	3.01 (± 0.4)	2.98 (± 0.3)	0.582	2.82 (± 0.3)	2.83 (± 0.3)	0.966

Data are presented as mean ± SD, *—P-value is statistically significant, T2DM—type 2 diabetes mellitus.

Young-Age Group (18–40 years): As illustrated in **Table 2**, participants with T2DM displayed elevated BMI, total body fat, VF, muscle mass, and bone mass compared to their non-T2DM counterparts; p-values <0.05. Conversely, they showed lower percentages of muscle and bone mass; p-value <0.05. Notably, this pattern persisted exclusively among young women when the data were analyzed by sex and age. In contrast, the young adult male group, non-T2DM individuals had a higher BMI compared to those with T2DM (32.10 vs. 29.48 kg/m²), with borderline significance (p = 0.05). Despite this, they exhibited a significantly higher muscle mass percentage (71.70 vs. 65.59) (p = 0.013), indicating a more favorable body composition despite the higher BMI.

Middle-Age Group (41–60 years): No significant differences in body composition were observed between individuals with and without T2DM in either sex (p-values > 0.05), refer to **Table 2**.

Older-Age Group (>60 years): Similarly, most body composition parameters did not differ significantly between individuals with and without T2DM. However, older women with T2DM had significantly higher BMI (p = 0.030) and visceral fat (p = 0.007) compared to their non-T2DM counterparts, as shown in **Table 2**.

## Discussion

This study highlights significant sex- and age-specific variations in body composition among individuals with T2DM. Our primary findings revealed that body composition differs notably by T2DM status in young women, with differences also seen in older women but to a lesser extent. These age-related differences did not manifest in middle-aged women or men in most age groups, except for a reduced muscle mass in young men with T2DM.

Previous studies have shown that individuals with T2DM tend to have higher body fat, particularly VF, compared to non-T2DM counterparts (15–18, 20, 21). Elevated VF is strongly associated with insulin resistance, a key factor in the development of T2DM, as it releases pro-inflammatory cytokines that disrupt insulin signaling and exacerbate metabolic dysfunctions (22–24). Our study, likewise, demonstrated a higher prevalence of obesity among individuals with T2DM but stands out by specifically examining the influence of sex and age on body composition, offering insights that previous studies have not fully explored (15–18, 20, 21).

Our findings indicate that hormonal and metabolic changes related to sex and age differentially may affect fat distribution and insulin sensitivity (24–26). Hormonal factors significantly influence body composition by affecting fat distribution and muscle mass. Estrogen tends to promote fat storage in women, while testosterone supports muscle growth in men (26). Consequently, women generally exhibit higher levels of body fat than men do, whereas men have greater muscle mass compared to women (26). Given this natural discrepancy, any additional fat accumulation in women tends to be more noticeable and may have unfavorable effects, potentially increasing the risk of developing T2DM. Conversely, the higher muscle mass typically observed in men may counterbalance some of the adverse effects of excess body fat, potentially attenuating differences in body composition between men with and without T2DM (23). Our study supports this concept, showing that lower muscle mass in young men may increase their susceptibility to T2DM, even if their body fat does not rise (27). This correlation might stem from skeletal muscles being a primary target of insulin action and representing a major site for insulin-mediated glucose uptake in the body (28). Interventions aimed at reducing VF and preserving muscle mass—such as resistance training and dietary modifications—are well-established strategies for preventing and managing T2DM. These interventions enhance insulin sensitivity, improve glucose metabolism, and help maintain a healthier body composition (29).

In our study, young women with T2DM exhibited higher absolute values for BMI, fat mass, muscle mass, and bone mass compared to non-T2DM controls, yet showed lower relative proportions of muscle and bone mass. While obesity increases all components of body composition due to greater mechanical loading and overall body size, individuals with T2DM—particularly when obesity is present—often experience a disproportionate increase in fat mass relative to muscle and bone. This altered composition may reflect a bidirectional relationship: excess adiposity contributes to insulin resistance and increases the risk of T2DM, while established T2DM may impair muscle growth and compromise bone quality through chronic hyperglycemia, insulin resistance, and inflammation (30, 31). Previous studies have shown that T2DM negatively affects bone metabolism, often reducing bone quality despite normal or elevated BMD (30, 31). The observation of this pattern in young women may relate to sex-specific differences in body composition and disease onset, as women typically have higher fat mass than men, and early-life obesity increases the risk of developing T2DM, especially in females, who tend to have higher body fat than males (24). These findings emphasize the importance of evaluating both absolute and relative body composition in metabolic risk assessment and highlight the need for further research to clarify these associations.

In the current study, older women with T2DM exhibited higher BMI and VF levels compared to non-T2DM women. The interplay between aging and menopausal hormonal shifts may act synergistically to exacerbate adverse changes in fat and muscle distribution. Both aging and menopause are characterized by an increase in fat mass and a decrease in muscle mass. These physiological changes not only contribute to increased adiposity but also reduce insulin sensitivity, thereby elevating the risk of developing T2DM (25, 32). Estrogen exerts direct protective effects on pancreatic β-cells and estrogen deficiency after menopause increases the risk of diabetes (24, 26). All women in the older group were postmenopausal, which likely contributed to the observed differences in adiposity between women with and without T2DM.

The absence of significant differences in body composition between middle-aged women with and without T2DM, in contrast to the younger and older groups, is intriguing and warrants further investigations. One possible explanation may be the relative stability in lifestyle habits—such as diet, physical activity, and healthcare engagement—that often characterizes this life stage, potentially buffering against more pronounced metabolic changes. Also, the 41–60 age range corresponds to the menopausal transition, during which some women may still be premenopausal or perimenopausal, while others may be postmenopausal, leading to heterogeneity in hormonal status that may obscure group-level differences. This variability in menopausal timing and progression could mask potential associations between T2DM and body composition in this cohort. Furthermore, it is possible that metabolic changes associated with T2DM in this group are more subtle or occur at a slower pace, becoming more apparent only in later life. As our study did not specifically assess menopausal status, hormone levels, or detailed lifestyle factors, future research should incorporate these variables to better understand the nuanced interplay between hormonal transitions, lifestyle, and body composition in middle-aged women with T2DM.

This study has several limitations. First, its cross-sectional design limits the ability to draw causal inferences. The relatively small sample size of young adults with T2DM is another limitation that may affect the generalizability of our findings. However, the presence of significant differences in this smaller subgroup suggests that obesity and elevated visceral fat accumulation among young women contributes to earlier T2DM onset. This pattern may reflect regional factors such as dietary shifts, sedentary lifestyles, and a genetic predisposition to central adiposity and insulin resistance in Saudi Arabian populations. While caution is needed when generalizing to other part of the world, the underlying mechanisms are likely relevant across populations and warrant further research in larger, more diverse cohorts. Furthermore, while we assessed exercise frequency, we did not use standardized frameworks of exercise, which may affect the comparability of our findings with other studies. Additionally, there is potential for recall bias in physical activity assessment, which may further limit the accuracy of this measure. Other factors, such as hormonal status, lifestyle habits (including dietary patterns), and genetic predispositions, were not examined, yet they could play significant roles in shaping body composition in individuals with T2DM. Future studies should incorporate longitudinal designs, standardized assessments of physical activity and dietary intake, and hormonal profiling to better understand the interplay between sex, age, and T2DM on body composition. It would also be valuable to stratify future analyses by menopausal status in women and testosterone levels in men to clarify hormonal contributions to observed trends.

In conclusion, this study contributes to the existing body of knowledge on the higher prevalence of obesity among individuals with T2DM, but stands out by demonstrating significant sex- and age-specific differences. These findings emphasize the need for tailored preventive and therapeutic strategies—for example, targeting adiposity in both young and older women, particularly those with elevated VF, and emphasizing muscle mass strengthening in young men and preservation in older men. Incorporating both age and sex into the design of interventions may lead to more effective and personalized approaches to the prevention and management of T2DM.

## Data Availability

The original contributions presented in the study are included in the article. Further inquiries can be directed to the corresponding author.
